# Outcomes of the Treatment of Head and Neck Sarcomas in a Tertiary Referral Center

**DOI:** 10.3389/fsurg.2015.00019

**Published:** 2015-05-19

**Authors:** Andrew Lindford, Benjamin McIntyre, Reginald Marsh, Craig A. MacKinnon, Charles Davis, Swee T. Tan

**Affiliations:** ^1^Wellington Regional Plastic, Maxillofacial and Burns Unit, Hutt Hospital, Wellington, New Zealand; ^2^Gillies McIndoe Research Institute, Wellington, New Zealand

**Keywords:** sarcoma, rhabdomyosarcoma, osteosarcoma, head, neck, survival, margins, flap

## Abstract

Head and neck sarcomas are a rare and heterogeneous group of tumors that pose management challenges. We report our experience with these tumors. Forty consecutive patients treated for 44 head and neck sarcomas between 1997 and 2014 were culled from our prospectively maintained head and neck database. Five patients were excluded. The adult cohort consisted 29 (83%) patients of a mean age of 57.7 years, with 33 sarcomas. The most common diagnoses were undifferentiated pleomorphic sarcoma (27%) and chondroblastic osteosarcoma (21%). Clear surgical margins were achieved in 24/33 (73%) lesions. Twenty-two patients received radiotherapy and/or chemotherapy. Fourteen patients developed local (*n* = 6), regional (*n* = 1) and distant (*n* = 7) recurrence. The overall 5-year survival was 66% with a mean survival interval of 66.5 months. Recurrent sarcoma, close (<1 mm) or involved surgical margins and advanced age were associated with statistically significantly reduced survival. The pediatric cohort consisted 6 (17%) patients, with a mean age of 9 years. Five patients had primary embryonal rhabdomyosarcomas and one had chondroblastic osteosarcoma. Clear surgical margins were achieved in five (83%) patients. All patients received adjuvant radiotherapy and/or chemotherapy. Mean survival interval was 102 months. Three patients developed local (*n* = 1) or distant (*n* = 2) recurrence. Twenty-three free and 8 pedicled flaps were performed in 25 patients. Eleven out of thirty-nine (28%) lesions in 11 patients developed a complication. In conclusion, head and neck sarcomas are best managed by a multidisciplinary team at a tertiary head and neck referral center and resection with clear margins is vital for disease control.

## Introduction

Four to ten percent of soft tissue and bone sarcomas in adults affect the head and neck region and account for 1–2% of all head and neck cancer (1–4). Head and neck sarcomas are a heterogeneous group of mesenchymal tumors of different histological subtypes. The complex anatomy of the head and neck region makes these tumors challenging to manage, particularly the need for multi-modality treatment and consideration for functional, esthetic, quality of life, and survival outcomes (5). Surgery remains the mainstay treatment for head and neck sarcomas, and histological subtype becomes a less critical determinant for survival than the operability in terms of anatomic considerations, tumor size, and stage at diagnosis (1).

As in tumors located elsewhere, positive surgical margins are a risk factor for treatment failure. However, wide surgical margins can be difficult to achieve due to the proximity of vital structures and tumor involvement of multiple closely related tissue planes. Furthermore, sarcomas tend to extend outside their pseudocapsule and invade tissues that may appear uninvolved during surgery.

This study presents outcomes of the treatment of head and neck sarcomas in our tertiary head and neck cancer referral center.

## Materials and Methods

Consecutive cases of head and neck sarcomas managed in the Head and Neck and Skull Base Program of the Wellington Regional Plastic Maxillofacial and Burns Unit between 1997 and 2014 were culled from our prospectively maintained head and neck database. Institutional ethics approval was obtained for this study. All patients who presented with a diagnosis of head and neck sarcoma were included in this study. Patients who declined treatment, those with tumors that were inoperable, or those did not undergo surgery as the primary treatment were excluded from the study. Patient demographics, tumor site, size and histologic type, types of ablative and reconstructive surgery, surgical complications, surgical margins, adjuvant therapies, local, regional, or distant recurrence, and overall survival were analyzed.

All patients were staged according to the American Joint Committee on Cancer guidelines for soft tissue and bone sarcomas (6, 7) and were managed by the multidisciplinary head and neck team. All ablative and reconstructive surgery were performed by plastic and craniomaxillofacial surgeons (STT, CAM, and CD), with involvement of a neurosurgeon when a transcranial procedure was required. A neck dissection was performed when there was clinical and/or radiological evidence of cervical nodal metastasis and/or tumor resection encroached on the neck, particularly if recipient vessels in the neck were needed in free flap reconstruction. All patients were routinely followed up by the multidisciplinary head and neck team, initially 1 month post-operatively and then 3-month with CT/MRI examination where indicated, for 5 years or until death.

Patients were divided into a pediatric cohort up to 16 years of age and an adult cohort >16 years.

### Statistical analysis

Statistical analysis of potential prognostic factors influencing survival was performed using SPSS v.22 (GraphPad Inc., La Jolla, CA, USA).

## Results

Of the 1,486 head and neck cancer patients, 40 (3%) patients with 44 sarcomas were identified. Five patients were excluded from further analysis because they declined surgery (*n* = 3), the tumor was inoperable (*n* = 1) and a 10-year-old child referred for reconstruction of maxillary hypoplasia following primary chemoradiotherapy for a right cheek rhabdomyosarcoma 7 years earlier. Thus, 29 (83%) adult and 6 (17%) pediatric patients were available for analysis (Table 1).

**Table 1:** **Patient demographics, tumor characteristics, and treatment modality**.

Characteristic	Number (%)
Age	
>16 years	29 (83%)
Up to 16 years	6 (17%)
Sex	
Female	18 (51.4)
Male	17 (48.6)
Primary tumor	33 (84.6)
Recurrent tumor	6 (15.4)
Head and neck subsite	
Skin and soft tissues	21 (53.8)
Facial skeleton	12 (30.8)
Skull base	6 (15.4)
Maximum diameter	
≤5 cm	24 (61.5)
>5 cm	15 (38.5)
Lymph node status	
Negative	3 (7.7)
Positive	36 (92.3)
Surgical margins	
Clear (>1 mm)	25 (64.1)
Close (<1 mm)	5 (12.8)
Involved	9 (23.1)
Treatment modality	
Surgery alone	12 (30.8)
Surgery + radiation	12 (30.8)
Surgery + chemotherapy	7 (17.9)
Surgery + radiation + chemotherapy	8 (20.5)

### Adult patients

#### Patient and Tumor Characteristics

**Figure 1:**
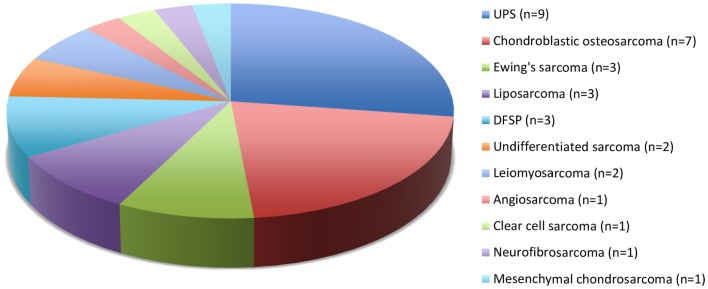
**Histological types and numbers of head and neck sarcomas**. UPS, undifferentiated pleomorphic sarcoma; DFSP, dermatofibrosarcoma protuberans.

Twenty-five European and 4 Maori adult patients with a mean age of 57.7 (median, 62; range, 23–92) years and of equal sex distribution, were treated for 33 sarcomas. One patient with Li–Fraumeni syndrome accounted for four asynchronous *de novo* sarcomas in the head and neck region (8). There were 21 (64%) cases of skin and soft tissue sarcomas of the head and neck and 12 (36%) sarcomas involved the facial skeleton (*n* = 9) and skull base (*n* = 3). Twenty-seven primary sarcomas and six recurrent sarcomas arose from the soft tissue (*n* = 22) and bone (*n* = 11), most commonly undifferentiated pleomorphic sarcoma (UPS) and chondroblastic osteosarcoma (Figure 1). The mean tumor size was 3.9 (range, 1–10) cm including stage I (*n* = 12), stage II (*n* = 5), stage III (*n* = 10), and stage IV (*n* = 6) disease at diagnosis. Two patients had distant metastases at presentation.

Surgical resection with curative intent was performed for 31 of the 33 sarcomas in 27 patients. Neck dissection was performed in six patients with osteosarcoma and one patient each with malignant peripheral nerve sheath tumor involving the vagus nerve, extra-skeletal Ewing’s sarcoma affecting the submandibular gland, leiomyosarcoma, liposarcoma, and UPS. Two patients were treated palliatively including one patient with Ewing’s sarcoma of the left maxilla with multiple lung metastases, and another with an 8 cm right mandibular osteosarcoma with a past history of stage IIIC ovarian cancer treated with surgery and chemoradiotherapy. Clear surgical margins were achieved in 24 (73%) sarcomas in 20 patients and this included close (<1 mm) surgical margins in four sarcomas while involved margins occurred in nine sarcomas. Selective neck dissection (*n* = 6) and a modified radical neck dissection (*n* = 5) were performed in 11 patients. Three of these patients had nodal involvement confirmed histologically: one case each for UPS, extra-skeletal Ewing’s sarcoma of submandibular gland, and osteosarcoma. Post-operatively 11 patients received radiotherapy alone, 5 received chemoradiotherapy and 6 received chemotherapy alone. Two patients declined radiotherapy and in two cases chemotherapy was discontinued due to toxicity.

#### Treatment Outcomes

Mean follow-up/survival interval was 66.5 (median, 35; range, 10–281) months. Overall 5-year survival was 66% for patients treated with curative intent. Local (*n* = 6), regional (*n* = 1), and distant (*n* = 7) recurrence occurred in 14 patients. The median interval to local recurrence was 14 months. Six of the seven patients with a local or regional recurrence had involved (*n* = 4) or close (<1 mm, *n* = 2) surgical margins. Four of these seven patients with loco-regional recurrence died from distant metastasis subsequently, two are alive and disease-free, and the remaining patient is alive but with widespread local disease. Overall, 11 (38%) patients died at a median interval of 21 months after treatment. All but one death occurred within 4.5 years, due to distant metastasis. The two patients treated palliatively died from distant metastases 15 and 36 months following treatment, respectively.

Recurrent sarcomas were associated with poorer survival than primary sarcomas (*p* = 0.01). In addition, close (<1 mm) or involved surgical margins (Fisher’s Exact test df = 28; *p* < 0.05) and advanced age (*p* < 0.05) were associated with reduced survival. There was no significant difference in survival between soft tissue and bone sarcomas, nor between the genders. Tumor size and tumor stage did not show statistical difference, but the subgroups were too small in numbers for reliable analysis.

### Pediatric patients

#### Patient and Tumor Characteristics

There were six pediatric patients with six primary sarcomas, with a mean age of 9 (median, 12; range 3–15) years, of equal sex distribution. There were five primary soft tissue sarcomas (all embryonal rhabdomyosarcomas), and one bone sarcoma (chondroblastic osteosarcoma) involving the skull base and facial skeleton. The mean tumor size was 6.3 (range 6.0–6.5) cm, stages III (*n* = 5) or IV (*n* = 1) at presentation.

Surgery with curative intent was performed in five patients, achieving clear surgical margins in all cases including close (<1 mm) margins in one patient. There was an involved surgical margin in the case treated palliatively, in which the tumor presented in the right pterygopalatine fossa with intracranial extension around the brainstem and cavernous sinus. Four patients received neoadjuvant chemoradiotherapy, one patient received radiotherapy alone and the remaining patient underwent neoadjuvant chemotherapy.

#### Treatment Outcomes

Median follow-up/survival interval was 74 (mean, 102; range, 13–252) months. One patient developed local and two patients developed distant metastasis, all of whom died within 31 months of treatment. The two patients who died from distant metastasis had close (<1 mm) or involved surgical margins. The case treated palliatively developed local recurrence 1 month following surgery and died 12 months later.

The number of pediatric cases was too small for meaningful statistical analysis of potential prognostic factors.

### Reconstructive procedures and surgical complications for the entire series

Twenty-three free flaps and eight regional flaps were performed in 25 patients (Table 2). The patient with Li–Fraumeni syndrome had five free flaps following resection of four asynchronous sarcomas. Another patient with an osteosarcoma involving the mandible also had five free flaps with no flap failure. A free fibular osseocutaneous flap and a superficial ulnar artery forearm flap were performed at initial surgery. Histology showed incomplete margins and a further resection and reconstruction with the contralateral free fibular osseocutaneous flap and superficial ulnar artery forearm flap was performed. Two additional free flaps were performed following flap failure for two other patients.

**Table 2:** **23 Free Flaps and 8 Regional Flaps in 25 Patients^a^**.

Types of flaps	Number
**Free flaps**	**23**
Rectus abdominis muscle or myocutaneous flap	6
Deep circumflex iliac artery flap	5
Fibula osteocutaneous flap	4
Lateral arm fasciocutaneous flap	3
Ulnar forearm fasciocutaneous flap	2
Superficial ulnar artery forearm flap	2
Superficial circumflex iliac artery fasciocutaneous flap	1
**Regional flaps**	**8**
Scalp transposition flap	3
Cervicofacial flap	2
Temporalis muscle flap	2
Pectoralis major myocutaneous flap	1

*^a^With 29 sarcomas*.

Eleven out of thirty-nine (28%) tumors in 11 patients developed 16 complications. More than one complication occurred in four of these patients. Eight patients with major complications required re-operation and the remainder were managed conservatively (Table 3). Three free flaps required a return to theater because of venous anastomotic thrombosis. Two of these flaps ultimately failed requiring a second free flap, both of which were successful. Hence complete flap loss occurred in 8% of the free flaps performed. There were no peri-operative deaths.

**Table 3:** **Complications in 11 patients**.

Complications	Number
**Major^a^**	**10**
Neck hematoma	4^f^
Free flap venous congestion^c^	3^f^
CSF leak following failed free flap^d^	1^f^
Infected methylmethacrylate	1
Wound dehiscence^e^	1
**Minor^b^**	**6**
Partial flap necrosis of cervicofacial flap	2
Chyle leak	1
Horner’s syndrome	1
Recurrent submental abscess	1
Neck seroma	1

*^a^Required re-operation*.

*^b^Managed conservatively*.

*^c^One salvaged, two required second free flap*.

*^d^Resolved with lumbar drain and a second free flap*.

*^e^Required wound debridement and direct closure*.

*^f^One complication each in a patient requiring one single re-operation*.

## Discussion

We show a 3% incidence of sarcomas among head and neck cancer cases treated at our center. The rarity and heterogeneity of histological subtypes affecting different age groups and different anatomic subsites make analysis of treatment outcomes of head and neck sarcomas challenging. In this study, the adult cohort consists of many histological subtypes whereas the pediatric group consisted of sarcomas involving the craniofacial skeleton, mostly rhabdomyosarcomas. In our series, adult sarcomas were six times as common as pediatric sarcomas, with UPS being the most prevalent (27%) subtype in adults, and rhabdomyosarcoma being the most prevalent subtype in children. This is in keeping with the recent SEER report on head and neck sarcoma (9).

Although both terms refer to the same clinical entity, there is now general agreement for the term *malignant fibrous histiocytoma* to be replaced with UPS (10). Being a regional plastic surgery center that also manages skin and soft tissue tumors may account for the higher proportion of UPS cases in our adult cohort compared with other series. We did not encounter radiation-induced sarcoma in our study. The absence of Kaposi sarcoma cases in our series reflects a low incidence of HIV infection and AIDS in New Zealand.

Rhabdomyosarcoma is the most common soft tissue sarcoma affecting patients younger than 20 years and occur most commonly (40%) in the head and neck region (11). In our study, bone-derived sarcomas represents 33% of cases with osteosarcoma being the most common subtype, followed by Ewing’s sarcoma, with the facial skeleton being the most common site. There is one case of extra-skeletal Ewing’s sarcoma involving the submandibular gland (12). We found no significant difference in survival between bone and soft tissue sarcomas.

The majority of sarcomas arise *de novo* with no identifiable causative factor. In our series, three patients had an associated predisposing condition. One patient had Li–Fraumeni syndrome, which is characterized by a p53 mutation, resulting in loss of tumor suppression. This predisposes the affected individual to developing soft tissue sarcomas, osteosarcoma, pre-menopausal breast cancer, brain tumors, and adrenocortical carcinoma (13). This patient developed two chondroblastic osteosarcomas, one liposarcoma, and one undifferentiated sarcoma over an 11-year period (8). The patient underwent curative resection of these asynchronous tumors and is disease-free during a 23-year follow-up. Another patient developed a chondroblastic osteosarcoma in the setting of monostotic fibrous dysplasia of the mandible. This patient underwent curative resection and is alive during a 9-year follow-up. The third patient with neurofibromatosis type 1 developed a malignant peripheral nerve sheath tumor arising from the left vagus nerve (14) and presented with a left vocal cord paralysis and central neck pain. Ablative surgery was performed and the patient is alive at 10-year follow-up.

Eighteen percent of adult cases in our series are recurrent sarcomas, which are associated with a statistically significant worse survival rate. Most of these cases were previously treated elsewhere without a multidisciplinary team involvement. This necessitates early referral to a tertiary head and neck cancer center for optimal management (15). Furthermore, the proximity to vital structures, their rarity and complexity in diagnosis and management of head and neck sarcomas underscore the need for referral of these patients to high-volume specialized centers to achieve optimal outcomes (16, 17).

While surgery remains the mainstay treatment for head and neck sarcomas, multi-modality treatment should be considered in all cases. The role of chemotherapy and/or radiotherapy is proven in rhabdomyosarcoma, Ewing’s sarcoma, and osteosarcoma (18–21). However, their precise role for many histological subtypes remains unclear. Rhabdomyosarcomas are chemosensitive and in most cases radical excision is considered after neoadjuvant chemoradiation.

Radiotherapy is often administered when surgery is unfeasible or in an adjunctive setting, either pre- or post-operatively, especially in high-grade tumors or in the presence of positive surgical margins. Earlier studies (3, 4) have shown that clear surgical margins are highly significant for local control and that the addition of adjuvant radiotherapy improves survival, over resection alone. Furthermore, post-operative adjuvant radiotherapy significantly increases local control over primary radiotherapy alone (60–82 vs. 43–50%, respectively) (22–24). There has been a long-standing debate regarding the role of pre-operative radiotherapy in the management of limb sarcomas (25–27) with one randomized study reporting improved survival at the expense of a greater risk of wound complications (25). Although theoretically pre-operative radiotherapy may increase the likelihood of achieving clear surgical margins in head and neck sarcomas, evidence remains lacking. Furthermore, significantly higher surgical complication rates following pre-operative radiotherapy have been demonstrated in patients undergoing treatment for head and neck cancer (28).

Generalizations about survival outcomes are difficult given the heterogeneity of the histological subtypes of the sarcomas affecting different age groups and different anatomical subsites. However, there are several key observations relating to the surgical treatment that are critical to the management of head and neck sarcomas. The ability to obtain clear surgical margins is a key determinant of disease control. Ablative surgery with clear surgical margins is usually achievable despite the anatomic constraints in the head and neck and skull base. Thirty-six of the 39 sarcomas (92%) were deemed to be operable with a curative intent. We achieved clear surgical margins in 73% of adult and pediatric sarcomas and our results confirm the importance of clear surgical margins on survival (20, 21, 29, 30). However, Zevallos et al. (31) show that positive surgical margins do not impact negatively on survival in pediatric patients undergoing surgical resection of sinonasal sarcomas (73% being rhabdomyosarcomas) following neoadjuvant chemotherapy.

The extent of invasion, especially intracranially, is a harbinger of inability to resect the tumor with clear margins. We usually only consider surgery for rhabdomyosarcoma if clear excision margins are feasible. Hence, in view of the associated morbidity and consequent impact on the quality of life we do not consider debulking surgery to be appropriate if the residual tumor will inevitably progress (32). In this study, we have achieved clear surgical margins in all rhabdomyosarcoma cases that were treated with curative intent, and a mean survival of 141 months. This compares favorably with other studies (9, 11). The patient treated palliatively was initially diagnosed as a Schwannoma. However, even if it had been a rhabdomyosarcoma, surgical resection would have been abandoned. Moretti et al. (33) have shown that involvement of the parameninges to be a poor prognosticator and orbital invasion to have a better prognosis, perhaps due to earlier detection and feasibility to achieve a clear surgical margin in the anterior cranial base.

Rahman et al. (34) observe that Ewing’s sarcomas abutting the infratemporal fossa are particularly challenging from a resection standpoint. However, a study by Givi et al. (35) demonstrates reasonable long-term survival following resection of infratemporal fossa tumors. Willers et al. (36) identify direct tumor extension into neurovascular structures, bones, continuous organs, or skin as risk factors for distant metastasis and death.

The feasibility of a curative resection often depends on the surgical morbidity and implications for the patient’s quality of life with regard to vision, speech, swallowing, and facial esthetics. We have shown that immediate flap reconstruction constitutes an important aspect of the surgical treatment of patients with head and neck sarcomas, many of whom require free flap transfers. Our 8% free flap failure rate is higher than the 6.3% overall institutional free flap failure rate (37), and is higher compared to a reported 3% failure rate of 133 free flap reconstructions following resection of head and neck sarcomas (38).

Our overall complication rate of 28% compares favorably with a published peri-operative complication rate of 30.1% and a late recipient site complication rate of 7.5% (38). Pre-operative radiotherapy in selected cases may be associated with fewer long-term complications and no effect on peri-operative complication rate (26, 27, 39).

Our 66% 5-year overall survival in adult sarcoma cases compares favorably with 44–70% reported in other series (3, 4, 9, 23, 24, 30, 36, 40–42). Long-term survival is likely with a follow-up of around 4.5 years being the critical landmark. All the deaths, except one, occurred before this time point. Advanced age is a significant risk factor for worse survival. Deaths usually results from hematogenous metastasis, usually to the lungs, brain, and bones (4, 36, 40). All deaths in our series resulted from pulmonary or visceral metastasis. We have previously shown that 3 of 11 patients with head and neck UPS presented with cervical metastasis (43). However, in this study, only one case of UPS had lymph node involvement confirmed histologically, and overall, three adult cases (9%) had lymph node involvement in comparison to 3% incidence reported by Brentz et al. (40).

## Conclusion

Head and neck sarcomas are a heterogenous group of tumors that pose significant management challenges. We have shown that surgical resection with clear margins is critical for survival and immediate reconstruction is an integral part of the surgical treatment. The high rate of surgical complications reflects the complexity of the clinical problem. Treatment of these tumors should be provided by multidisciplinary teams at specialized centers to achieve optimal outcomes.

## Conflict of Interest Statement

The authors declare that the research was conducted in the absence of any commercial or financial relationships that could be construed as a potential conflict of interest.
